# Nebulized Hybrid Nanoarchaeosomes: Anti-Inflammatory Activity, Anti-Microbial Activity and Cytotoxicity on A549 Cells

**DOI:** 10.3390/ijms26010392

**Published:** 2025-01-04

**Authors:** Sofia Giuliana Guerin Stabile, Noelia Perez, Horacio Emanuel Jerez, Yamila Roxana Simioni, Estefanía Butassi, Martin Daniel Mizrahi, Matias Leonardo Nobile, Ana Paula Perez, Maria Jose Morilla, Leticia Herminia Higa, Eder Lilia Romero

**Affiliations:** 1 Centro de Investigación y Desarrollo de Nanomedicinas (CIDeN), Departamento de Ciencia y Tecnología, Universidad Nacional de Quilmes, Roque Sáenz Peña 352, B1876 Bernal, Argentinaapperez@unq.edu.ar (A.P.P.);; 2 Facultad de Ciencias Biológicas y Farmacéuticas, Universidad Nacional de Rosario, Suipacha 531, S2000 Rosario, Argentina; ebutassi@fbioyf.unr.edu.ar; 3 Instituto de Investigaciones Fisicoquímicas Técnicas y Aplicadas (INIFTA), Departamento de Química, Facultad de Ciencias Exactas, Universidad Nacional de La Plata CCT La Plata—CONICET, Diagonal 113 y 64, B1900 La Plata, Argentina; mizrahi@fisica.unlp.edu.ar; 4 Facultad de Ingeniería, Universidad Nacional de La Plata, Calle 1 esq. 47, B1900 La Plata, Argentina; 5 Laboratorio de Biocatálisis y Química de Ácidos Nucleicos (LABiQAN), Universidad Nacional de Quilmes, Roque Sáenz Peña 352, B1876 Bernal, Argentina; mnobile@unq.edu.ar

**Keywords:** nanoarchaeosomes, biogenic silver nanoparticles, antitumoral, antimicrobial

## Abstract

The properties of two hybrid nanoarchaeosomes (hybrid nanoARCs) made of archaeolipids extracted from the halophilic archaea *Halorubrum tebenquichense* and combining the properties of archaeolipid bilayers with metallic nanoparticles are explored here. BS-nanoARC, consisting of a nanoARC loaded with yerba mate (*Ilex paraguariensis)* extract (YME)-biogenic silver nanoparticles (BSs), and [BS + BS-nanoARC], consistent of a BS-nanoARC core covered by an outer shell of BSs, were structurally characterized and their therapeutic activities screened. By employing 109 ± 5 µg gallic acid equivalents (GAEs) and 73.4 µg chlorogenic acid/ YME mg as a silver reductive agent, spherical, heterogeneously sized (~80 nm diameter), −27 mV ζ potential, 90% Ag^0^ and λ_max_ 420 nm BSs were obtained. We further prepared ~100–200 nm diameter, −57 mV ζ potential BS-nanoARC and ~300 nm diameter, −37 mV ζ potential [BS + BS-nanoARCs]. Freshly prepared and nebulized BS-nanoARCs reduced the release of TNF-α, IL-6 and IL-8 by LPS-irritated THP-1-macrophages and were highly anti-planktonic against *S. aureus* (MIC_90_: 13 ± 0.8 µg Ag/mL). While the nanoARCs and BS-nanoARCs were innocuous, freshly prepared [BS + BS-nanoARCs] magnified the cytotoxicity of BSs (IC_50_ 12 µg Ag/mL vs. IC_50_ ~36 µg Ag/mL) on A549 cells. Such cytotoxicity remained after 30 days in the dark at 4 °C, while that of BSs was lost. Freshly prepared BSs also lost activity upon nebulization, whereas freshly prepared [BS + BS-nanoARCs] did not. However, the cytotoxicity of the [BS + BS-nanoARCs] was also lost when nebulized after 30 days of storage. Despite the harmful effects of storage and mechanical stress on the structure of the more active [BS + BS-nanoARCs], hybrid nanoARCs are promising examples of nanomedicines combining the properties of archaeolipids with antimicrobial silver nanoparticles and anti-inflammatory polyphenols that could complement oncologic therapies, reducing the usage of classical antitumoral agents, corticosteroids, and, importantly, of antibiotics, as well as their waste.

## 1. Introduction

Nanoarchaeosomes (nanoARCs) are lipid nanovesicles made of archaeolipids extracted from microorganisms from the archaea domain [1]. NanoARCs prepared in this work are made of *sn* 2,3 ether-linked phytanyl saturated polar archaeolipids, extracted from the halophilic archaea *Halorubrum tebenquichense* added with bacterioruberin (*all-trans BR*), a highly antioxidant xanthophyll with 36 Å length membrane-spanning fully unsaturated isoprenoid chain that inserts transversally into bilayers, potentially protecting cells from mitochondrial ROS [2,3]. Unlike soy phosphatidylcholine liposomes, nanoARC bilayers resist hydrolysis, oxidation and the activity of stereospecific phospholipases. Polar archaeolipids are rich in the methyl-branched chain archaeolipid phosphatidyl glycerophosphate methyl ester (PGP-Me), a natural ligand for cells expressing the scavenger receptor class A (SRA) [4]. SRA1 is involved in the extensive internalization of nanoARCs, and is responsible for a further magnification of the activity of cargoes targeting the endosomes [5].

Silver nanoparticles are widely acknowledged as antimicrobial agents due to their large volume of commercial applications [6]. The antimicrobial activity of silver nanoparticles, industrially synthesized with inorganic, usually toxic reducers [7,8], is thought to be mediated by their preferential binding to key enzymes for bacterial metabolism, where the oxidation of Ag^0^ to Ag^+^ in the vicinity of the targets would induce oxidative stress in bacteria in addition to inflammation in mammalian cells [9,10]. Using natural extracts as Ag^+^ reducing agents to produce biogenic nanoparticles [11] has considerably expanded the portfolio of metallic nanoparticles. Being less toxic than their inorganic counterparts, biogenic silver nanoparticles (BSs) can be prepared in different sizes, shapes and surface features, made of molecules used as reducing agents [12,13]. BSs are good candidates for reducing the indiscriminate use of antibiotics on surficial infections [14]. Counting on agents to replace classical antibiotics would aid in controlling the emergence of bacterial resistance and reduce the agro-ecosystem contamination caused by antibiotic residues coming from animal husbandry or the improper disposal of medicinal products [15].

*Less explored is the ability of inorganic and biogenic silver nanoparticles to display antitumoral activity, which has been screened neither* in vitro *or* in vivo models [16,17,18]. Antitumoral activity requires delivering nanoparticles to eukaryotic cells from deep organ microenvironments. *Their biodistribution and pharmacodynamics could be upgraded if loaded into larger nanostructures such as nanovesicles, whose surface and size can be endocytosed by eukaryotic cells. The targeted delivery of silver nanoparticles to cells, thus, may magnify their activity.*

Non-small cell lung cancer (NSCLC) is the most common form of lung cancer [19], and its complex treatment is based on surgery, chemotherapy, radiotherapy, targeted therapy or immunotherapy [20]. We speculate that the antitumoral activity of BSs could be improved by increasing either their intracellular and/or lysosomal delivery, since the cytotoxicity of silver nanoparticles on A549 cells (used to model NSCLC [20]) has been reported to be mediated by lysosomal damage [21]. Chemotherapy usually induces neutropenia and favors the installation of concurrent infections, including Gram + bacteria such as *Staphylococcus aureus* [22]; likewise, the response to treatments may be influenced by the tumor microbiome, known to have a prevalence of enteric and potentially pathogenic bacteria, which includes the genus *Pseudomonas* [23]. Currently, mainly Gram—infections of the lung are treated systemically; nonetheless, the access of antibiotics to the lung parenchyma is difficult [24]. In addition, the efficacy of lung oncologic therapies could be enhanced by agents acting on infected surfaces that combine antimicrobial and antitumor activities. Because of their size between ~25 and 500 nm diameter, nanomedicines can neither cross epithelial nor endothelial walls and must be administered intravenously to target inner organs [25,26]. However, mucosal surfaces can be reached by instillation or inhalation, two administration routes that offer fewer risks and higher patient adherence to treatment.

The industrial manufacture of nanovesicles, despite being established, remains technical and economically challenging [27,28,29,30,31]; only those agents offering a remarkable therapeutic improvement are worth it. In this work, hybrid (combining metal and lipids), green (based on natural products: plant and microorganism extracts) nanomedicines lacking small antitumoral drugs, antibiotics or corticosteroids in their composition, were prepared as follows: *yerba mate extract (YME) was used as a reducing agent of Ag^+^ to yield BSs and BSs were loaded into nanoARCs, which, after the addition of BSs, rendered [BS + BS-nanoARC] a more voluminous nanoparticle.* Determining the performance of hybrid nanoARCs on in vitro cell models is the first critical step in selecting the most promising (stable and active) formulation. The nanoARC structure could protect silver nanoparticles against oxidation, and mechanical or photodamage suffered during storage. NanoARCs, however, are not pH-sensitive, and, paradoxically, their internalization may reduce the activities of voluminous cargoes such as BSs by impairing their access to extra-endo-lysosomal targets, such as the nuclei of cell cytoplasm [32]. Because of this same reason, the delivery of BSs to extracellular bacteria and fungi may also be compromised. Assessing the effect of BSs or hybrid nanoARCs on A549 cells before and upon nebulization or storage, together with their anti-inflammatory, antimicrobial and antioxidant activities, will clarify their potential as therapeutic agents. This is the first study to examine the effect of nebulized highly complex hybrid nanovesicles that combine halophilic archaeolipids with biogenic silver nanoparticles on A549 cells. Here, the importance of studying their performance after being stored or after they have been subjected to significant shear stress is highlighted.

## 2. Results

### 2.1. Structural Characterization of BSs

The structural features of BSs prepared by the sequential reduction of AgNO_3_ by adding YME solution (Figure S1A,B) are shown in Table 1.

**Figure 1:**
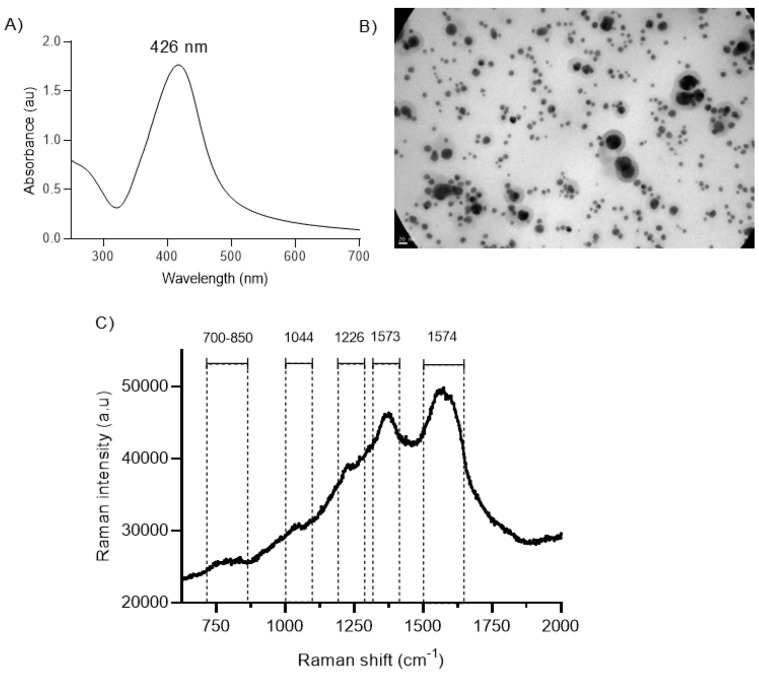
(**A**) UV–Vis spectra of BSs; (**B**) TEM of BSs (150,000×); (**C**) SERS spectrum of BSs.

The UV–Vis spectra of the BSs displayed the typical plasmonic peak at 420 nm, corresponding to spherical nanoparticles (Figure 1A). Nearly 90% of the Ag was Ag0, as determined by XR absorption (Figures S2 and S3). A representative TEM micrograph of heterogeneously sized BSs, with metal cores surrounded by a thick diffuse layer, probably corresponding to the oxidized components of the YME, is shown in Figure 1B. Figure 1C shows the Raman spectra of the BSs. The surface plasmon confinement in the BSs magnified the Raman signals of molecules close to the nanoparticle surface, inducing surface-enhanced Raman scattering (SERS) phenomena [33]. According to previous assignments on the SERS signals of CGA on silver nanoparticles [34], the BS spectrum showed typical strong bands at 1373 cm^−1^ (CO_2_ sym. str., cyc.CH, COH bend, cyc. ring CH bend, C_24_O_27_H_42_ bend) and 1574 cm^−1^ (ph. ring str., C1=C2 str., C-ph. str.). Weak bands were also observed at 1226 (ph. ring CH, COH bend), 1044 and between 700 and 850 cm^1^ [eth: ethylene; ph: phenyl ring; cyc: cyclohexane ring; s: strong; m: medium; w: weak; sh: shoulder; str.: stretch].

### 2.2. Structural Characterization of Hybrid nanoARCs

**Figure 2:**
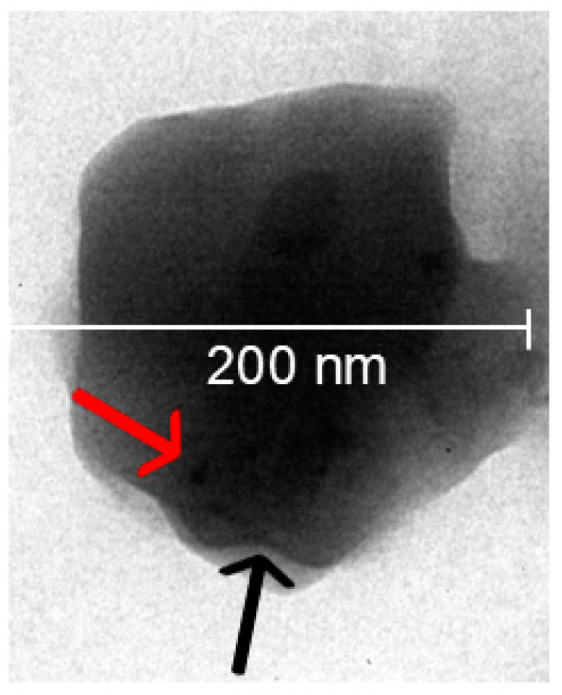
Representative TEM of BS-nanoARC (140,000×). BS: 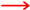; lipid bilayer: 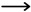.

BS-nanoARCs prepared by hydrating lipid films with 1 mL BS (1 mg Ag/mL) suspension were seeded in a Sephadex G 50 spin column to wash the unloaded BSs. Nearly all phospholipids from the nanoARCs were eluted in fractions (F) 1 and 2. When seeding the BSs, the plasmonic peak was not detected up to F15, meaning the BSs remained trapped in the column. The size and ζ potential of the eluted BS-nanoARCs are shown in Table 1. The broad plasmonic peak from the BSs overlaps with the BR peaks (at 385, 405, 455–460, 490 and 525 nm), impairing our ability to quantify the amount of BSs in the BS-nanoARCs by measuring the intensity of the plasmonic peak in the UV–Vis spectrum. Hence, the content of BSs in the nanoARCs was screened by seeding the eluted BS-nanoARCs on a TLC plate. The BSs, unlike previously reported [35,36], did not run on the TLC plate but were stacked at the seeding point as a grey spot (Figure S4). The grey spot was present in the seeded eluted BS-nanoARCs, suggesting that at least part of the BSs were loaded into the nanoARCs instead of stacked in the Sephadex column. The encapsulation percentage of Ag in the eluted BS-nanoARCs was further estimated by XANES and EXAF, resulting to be ~90% (Figure S2). The resulting BSs were submicronic in size, polydisperse, with a Z average of ~80 nm, and had a highly negative ζ potential of ~−28 mV. The size and polydispersity index of the BS-nanoARCs were slightly larger than the BSs. The size of the [BS + BS-nanoARCs] was nearly two times higher than that of the BS-nanoARCs, accompanied by an increase from −58 to ~−38 mV in ζ potential, suggesting that the BSs were partitioned on the BS-nanoARCs’ surface. BSs reduced the GP of the BS-nanoARCs (compared to nanoARCs alone), but did not affect the micro-viscosity (measured as FA) of the nanoARC bilayers. The reduced GP, indicative of high disorder [37], may be caused by the BSs’ partition into the nanoARC bilayers. Figure 2 shows a representative TEM image of a BS-nanoARC, where dark areas compatible with inner BSs have been pointed out. As shown in Table 2, the antioxidant activities measured by ABTS of the BS-nanoARCs and of BSs were comparable with that of Trolox (IC_50_ ~4.3 ± 0.3 µg/mL). The antioxidant activity of the YME was low and showed poor reproducibility.

### 2.3. Cytotoxicity on THP-1 Macrophages and Effect on Lysosomes

The IC_50_ of the BSs and BS-nanoARCs on THP-1 macrophages are shown in Table 3.

**Figure 3:**
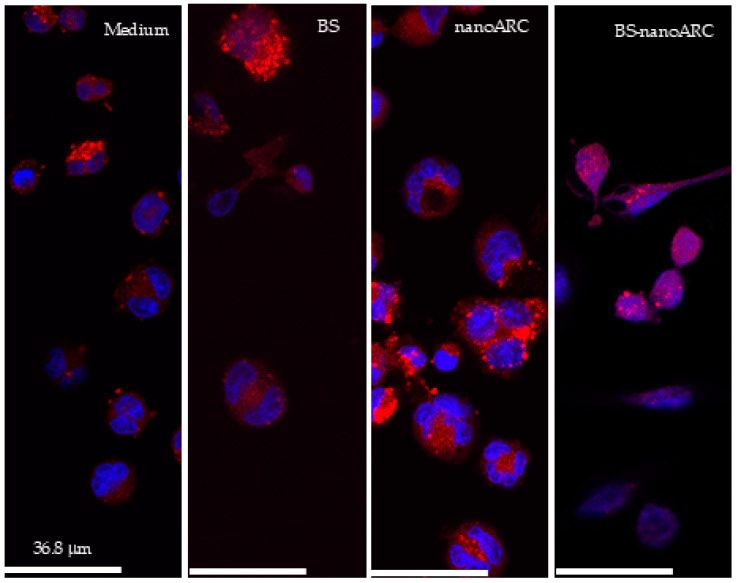
Confocal fluorescence images of THP-1 macrophages stained with Lysotracker (red) and Hoechst (blue) after incubation with medium, BSs, nanoARCs and BS-nanoARCs. Magnification 63×.

Figure 3 shows representative images of the effect of endocytosis of BS-nanoARCs on THP-1 cells’ acid vessel morphology. Lysotracker (LR) is an acidotropic dye that non-specifically stains acidic compartments (such as the increased lysosomal mass occurring during autophagy, or autophagosomes, even if autophagic flux is blocked). The LR fluorescence may indirectly quantify the degree of autophagy, since its increase follows that of the autophagy marker LC3II [38]. A rapid loss of LR fluorescence, on the other hand, occurs after losing lysosomal integrity, and lysosomal proteases are released into the cytosol [39,40,41]. Opposite to cationic nanoparticles, nanoARCs (having highly negative ζ potential), do not cause lysosomal damage via the sponge effect. As previously reported [42], the endocytosis of nanoARCs induced brilliant enlarged cytoplasmic vessels, compatible with lysosomal enlargement, suggesting autophagy induction [38]. Instead, the endocytosis of BS-nanoARCs induced morphological changes and a significant loss of lysosomal acidity. The intake of BSs did not cause observable modifications in lysosomes. Figure S5 shows a representative selection of changes induced by the BSs and BS-nanoARCs in A549 cells. They were much less evident, without lysosomal alterations, probably due to less extensive internalization than in macrophages.

### 2.4. Anti-Inflammatory and Anti-ROS Activity on THP-1 Macrophages

**Figure 4:**
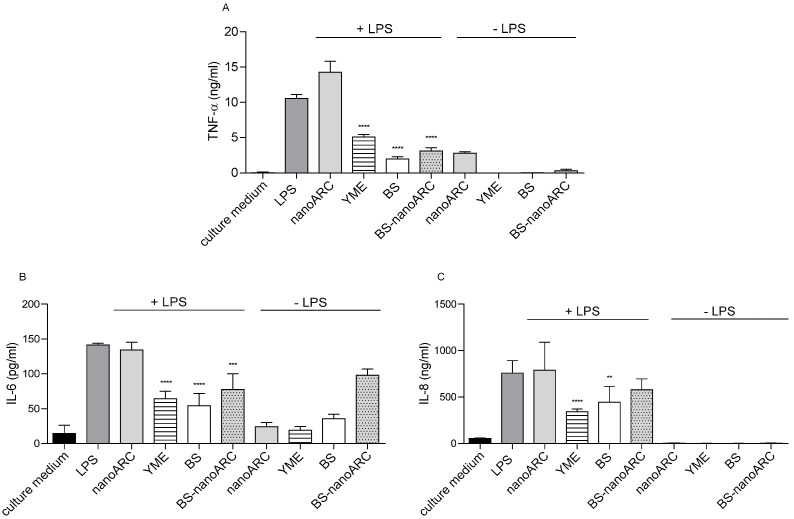
Pro-inflammatory cytokines released after uptake by THP-1-derived macrophages. (**A**) TNF−α; (**B**) IL−6; (**C**) IL−8. Statistical significance compared with LPS was determined using a Kruskal– Wallis non-parametric test followed by Dunn’s multiple comparisons, ** *p* < 0.01; *** *p* < 0.001; **** *p* < 0.0001.

**Figure 5:**
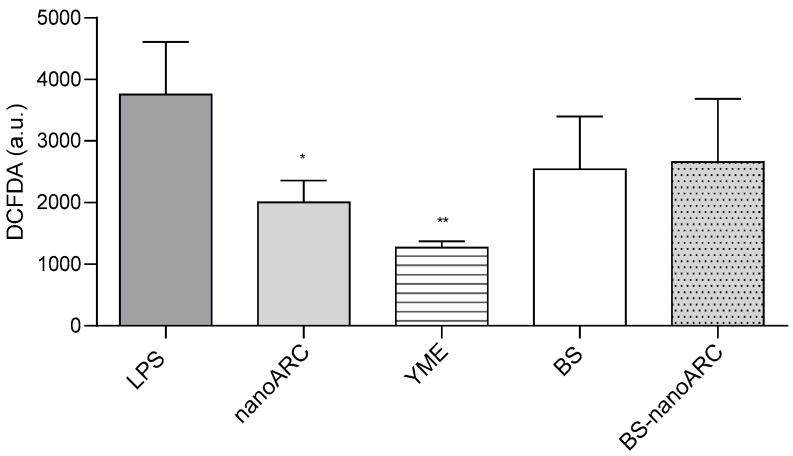
Antioxidant activity on THP– 1 macrophages. Statistical significance compared with LPS was determined using a Kruskal– Wallis non-parametric test followed by Dunn’s multiple comparisons, * *p* < 0.05 and ** *p* < 0.01.

The anti-inflammatory and antioxidant activities of (50 µg PL/mL, 5 µg Ag/mL) BS-nanoARCs are shown in Figure 4 and Figure 5, respectively.

5 µg Ag/mL BSs, 40 µg YME/mL and BS-nanoARCs reduced the release of cytokines from THP-1 macrophages irritated with LPS. The anti-inflammatory activity could be attributed to the YME on the BSs’ surface. On the other hand, the antioxidant BR and the polar archaeolipids neither improved nor influenced the anti-inflammatory profile of the formulations. Only (50 µg PL/mL) nanoARCs and (40 µg/mL) YME displayed anti-ROS activity.

### 2.5. Antimicrobial Activity

#### 2.5.1. Anti-Planktonic Activity

As shown in Table 4, the BSs exhibited anti-planktonic activity on *S. aureus* and PAO1 at ~12.5 µg Ag/mL and 6.4 µg Ag/mL (values in the order of those from other silver nanoparticles [11]), respectively. BS-nanoARCs also displayed anti-planktonic activity against *S. aureus*, but their activity against PAO1 was ~8 times lower than BSs. The anti-planktonic activity of the BSs and BS-nanoARCs is comparable to that of the newer antibiotics currently used against PA01 and S aureus infections: the MICs of colistin, ceftazidime–avibactam (a combination of ceftazidime, a third-generation antipseudomonal cephalosporin, with avibactam, a novel beta-lactamase inhibitor) and amikacin against *PA01* strains isolated from patients are 2, 4 and 8 µg/mL, respectively [43]. Likewise, the MIC of vancomycin, azithromycin and rifampicin against *S aureus* ATCC 25,923 in different culture media oscillate between (1–4), 1 and (0.115–0.03; 0.0039–0.031) µg/mL, respectively [44]. This highlights the feasibility of nebulizing nanoparticulate formulations as an alternative to classical treatments.

#### 2.5.2. Anti-Biofilm Formation Activity

As shown in Table 4, BSs impaired biofilm formation at 6 µg/mL and 7.5 µg/mL against *S. aureus* and PAO1, respectively. In contrast, the activities of the BS-nanoARCs were 14 times lower than BSs.

#### 2.5.3. Antifungal Activity

As shown in Table 5, BSs also presented the maximal anti-Candida activity (MIC 12.5 µg/mL, MFC 25 µg/mL). Again, the activity of the BS-nanoARCs was four times lower than that of the BSs and at the expense of a high (>250 µg/mL) phospholipid concentration.

### 2.6. Cytotoxicity on A549 Cells

The IC_50_ values of BSs and hybrid nanoARCs are shown in Table 6.

**Figure 6:**
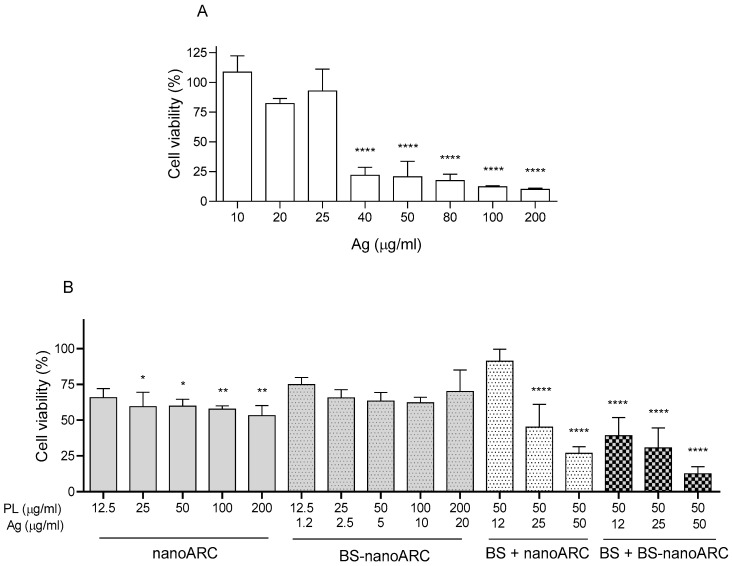
A549 cell viability after 72 h when treated with (**A**) BSs and (**B**) hybrid-nanoARCs. Statistical significance compared with medium control was determined using a Kruskal– Wallis non-parametric test followed by Dunn’s multiple comparisons, * *p* < 0.05, ** *p* < 0.01; **** *p* < 0.0001.

Figure 6 shows that between ~1 and 20 µg Ag/mL, with 12.5–200 μg PL/mL as an upper limit [45], the BS-nanoARCs were not cytotoxic (IC_50_ > 20 µg Ag/mL, >200 µg PL/mL). The cytotoxicity of [BS + BS-nanoARCs] instead tripled that of the BSs (IC_50_~12 µg Ag/mL vs. IC_50_~36 µg Ag/mL). Remarkably, the IC_50_~30 µg Ag/mL of BS + nanoARCs used as a control was close to that of the BSs. This suggests that the cytotoxicity on A549 cells cannot be attributed to a single agent (either BS or BS-nanoARC internalization), but rather to a structural combination, such as the one offered by the voluminous [BS + BS-nanoARC].

**Figure 7:**
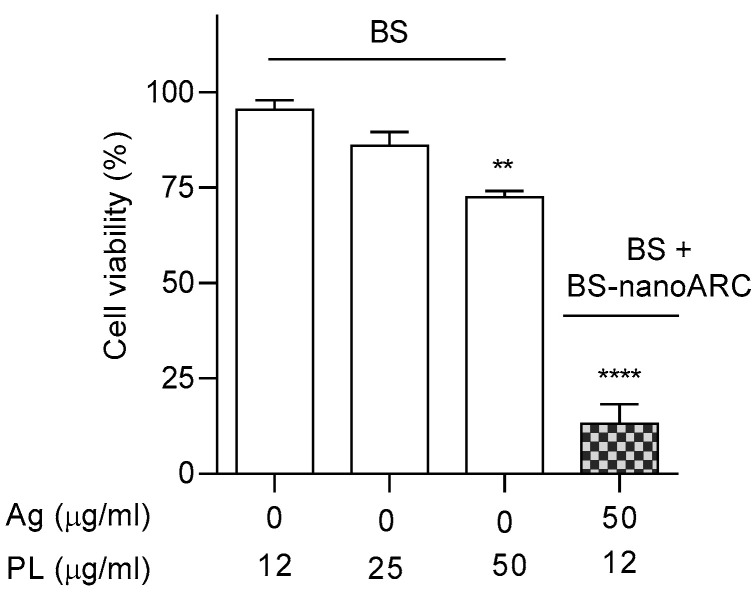
A549 cell viability after 72 h when treated with nebulized BSs and BS + BS-nanoARCs. Statistical significance compared with medium control was determined using a Kruskal– Wallis non-parametric test followed by Dunn’s multiple comparisons, ** *p* < 0.01; **** *p* < 0.0001.

After nebulization, the BSs maintained their size and plasmonic peak, and nearly 90% of the silver was recovered. The size and Ag/lipid ratio of the hybrid nanoARCs were also kept, despite the PL recovery being between 40 and 30%, as shown in Table 7. The cytotoxicities on A549 cells of the nebulized BSs and hybrid nanoARCs are shown in Figure 7. Upon nebulization, the cytotoxicity of the BSs was lost, while the [BS + BS-nanoARCs] remained cytotoxic (despite their structure consisting partly of surficial associations).

**Figure 8:**
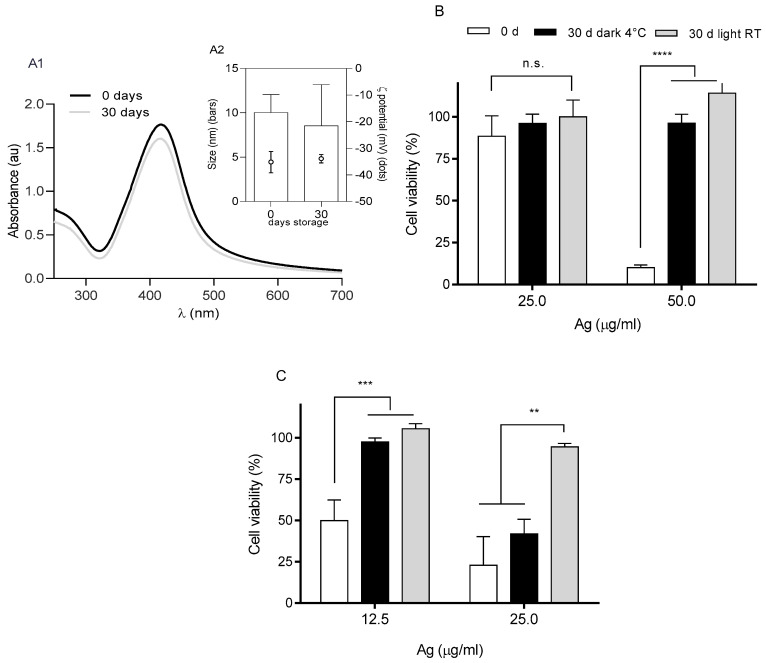
Stability upon storage: (**A1**) plasmonic peak and (**A2**) size of BSs. A549 cell viability after treatment with stored (**B**) BSs and (**C**) [BS + BS-nanoARCs] (50 μg PL/mL, 12.5–25 μg total Ag/mL). Statistical significance compared with medium control was determined using a Kruskal–Wallis non-parametric test followed by Dunn’s multiple comparisons, ** *p* < 0.01; *** *p* < 0.001; **** *p* < 0.0001.

The cytotoxicity on A549 was determined for fixed doses of 25 and 50 µg Ag/mL BSs, and 50 µg PL/mL, 12.5 or 25 µg total Ag/mL [BS + BS-nanoARCs] after being stored for 30 days either at 4 °C in the dark or at RT in the light, as shown in Figure 8. As expected, light exposure eliminated the plasmonic peak of the BSs together with their cytotoxicity at 25 or 50 µg Ag/mL on A549 cells. In the dark, their colloidal stability, ζ potential and plasmonic peak were maintained (Figure 8(A1,A2)), but their cytotoxicity was also lost (Figure 8B). [BS + BS-nanoARCs] also lost their cytotoxicity after the light exposition, but their cytotoxicity at 25 µg total Ag/mL after being stored for 30 days at 4 °C in the dark was preserved (Figure 8C). When formulated as a [BS + BS-nanoARC], the cytotoxicity of the BSs was magnified and could be preserved for at least 30 days at 4 °C in the dark.

When the BSs or [BS + BS-nanoARCs] were nebulized after being stored for 30 days in the darkness at 4 C, both lost their cytotoxicity; the latter enlarged their size above 1 µm diameter (Table 7).

The antioxidant, anti-inflammatory and antimicrobial activity of the BSs and BS-nanoARCs were maintained after being stored for 30 days at 4 °C in the dark; again, the activities of the nebulized BS-nanoARCs remained only if freshly prepared.

## 3. Discussion

Most reports about the effect of nanovesicles on A549 cells do not refer to their challenging delivery to the deep respiratory tract. While aerosolization is the most direct access route to the lungs, the structure of the particulate matter can be destroyed by the shear forces of nebulization, or clog the mist outlet [46]. Nebulized liposomes, for instance, must be tailored in composition to resist the strain stress of nebulization, usually adding costly components such as cholesterol or hydrogenated lipids [47]. As well as being naturally targeted to SRA1 [4] and displaying high colloidal stability due to their high negative ζ (responsible for their small sizes) [48], nanoARCs prepared with lipids extracted from halophilic archaea such as *H. tebenquichense* are also mucopenetrant [49] and known to tolerate nebulization stress [4], constituting optimal nanovesicles to target cells in the deep lung.

As pointed out above, the antimicrobial effect of biogenic silver nanoparticles prepared with yerba mate extract or its waste [40,41,42,43,44,45,46,47,48,49,50,51,52,53] has been reported in the search for eco-friendly materials and processes to replace antibiotics and reduce the emergence of resistance. Aside from their use as antimicrobial agents, few studies are currently available addressing the effect of silver nanoparticles on A549 cells. Such effects are specific to the structural features of each silver nanoparticle. For example, ~10 μg Ag/mL PVP-coated silver nanoparticles induce ~50% cytotoxicity [54]. Additionally, 20 nm diameter citrate-coated silver nanoparticles cause lysosomal dysfunction and cell death at 100 or 200 μg Ag/mL for 24 h [55]. An IC_50_ of 4.5 µg Ag/mL has recently been reported for ~17.3 nm sized, −28.5 mV ζ potential biogenic silver nanoparticles prepared using the root extract of *Reynoutria japonica* Houtt as a reducing agent [21]. With an IC_50_ of ~36 μg Ag/mL, the BSs prepared here displayed intermediate cytotoxicity on A549 cells. We speculate that the structural features of the nanoARCs comprising hybrid nanoARCs would protect/magnify the therapeutic activities of these BSs. While antimicrobial, anti-inflammatory and antioxidant activities were only determined for the BS-nanoARCs, the cytotoxicity of each hybrid nanoARC against A 549 cells was surprisingly different: [BS + BS-nanoARCs] with ~12 μg total Ag/mL distributed partly within the (BS-nanoARC) core and partly on the core surface as a BS shell reduced cell viability by ~50%, tripling the original cytotoxicity of the BSs. Opposingly, neither BS-nanoARCs with 10 μg total Ag/mL within its inner space, nor (BS+nanoARCs) with ~12 μg total Ag/mL outside the nanoARC, induced cell cytotoxicity. The cytotoxicity of [BS + BS-nanoARCs] on A549 cells seemed to be related to the distribution of BSs on the nanoARC structure; its internalization as a whole nanoparticle and not of its separated components would be the key to magnifying BS cytotoxicity on A549 cells.

The first steps of preclinical development of new complex nanoparticles such as BSs and hybrid nanoARCs require assessing their activities either freshly prepared or after storage, and before and after nebulization stress. Remarkably, it was found that despite their size and plasmonic peak remaining unchanged, the BSs had lost their cytotoxicity after being stored for 30 days in darkness at 4 °C. In the same conditions, [BS + BS-nanoARCs] remained cytotoxic, showing that this voluminous hybrid nanoARC could partly preserve the cytotoxicity of BSs. Neither polar archaeolipids, with saturated and branched structures useful to protect and dissolve different small molecules [1], nor the antioxidant BR, however, protected the BSs against chemical and photodegradation, since their cytotoxicity vanished after light irradiation.

The other remarkable finding was that although their size and plasmonic peak remained unchanged, the BSs lost cytotoxicity after nebulization. The activity of [BS + BS-nanoARCs] instead endured nebulization but vanished if nebulized after being stored for 30 days in the dark at 4 °C. That fact suggested that the complex association between BSs and nanoARC bilayers was modified upon storage, probably by outer BS detachment.

Ours is also the first report to show the effect of biogenic silver nanoparticles prepared with YME on THP-1 macrophages, a cell line expressing SRA1 [56] and capable of extensively internalizing nanoARCs. Our results showed that the cytotoxicity of BSs (IC_50_~22 µg Ag/mL) was tripled by BS-nanoARCs (IC_50_~7 µg Ag/mL, 70 µg PL/mL). Such a result agrees with the increased cytotoxicity (at a non-reported lipid concentration) of liposomal ~20 nm borohydride silver nanoparticles vs. that of free silver nanoparticles, reported previously [57].

As for the anti-inflammatory effect, we found that freshly prepared BSs and BS-nanoARCs were equally anti-inflammatory on THP-1 macrophages stimulated with LPS. Macrophages internalize nanoARCs at a high uptake rate mediated by SRA1 [58], delivering their cargo to the endo-lysosomal system [59], in this case, an acidic medium where BSs would be rapidly dissolved [60]. The available data on silver nanoparticles’ effect on inflammation, on the other hand, are extremely variable. Inorganic borohydride silver nanoparticles, for instance, are reported to be pro-inflammatory, but became anti-inflammatory on THP-1 macrophages when loaded into ~130 nm extruded DPPC liposomes [61], while other authors describe silver nanoparticles as strongly anti-inflammatory [62]. Similarly, between 50 and 100 µg Ag/mL, most inorganic or polysaccharide-coated silver nanoparticles [63] are described to increase intracellular ROS levels. Here, 5 µg Ag/mL BSs or BS-nanoARCs did not modify the intracellular ROS levels. On the other hand, the expected anti-ROS activity of BR, mediated by enzymatic mechanisms affecting the redox state observed after cell internalization, was not revealed by cell-free radical cation screening such as the ABTS technique [64]. The absence of the anti-ROS activity of BS-nanoARCs may be explained by the consumption of BR by the BSs within nanoARCs. This is supported by the fact that nanoARCs (lacking BSs), effectively decreased the intracellular ROS levels. In coincidence with previous reports on other biogenic silver nanoparticles [21], the cell-free antioxidant activity measured by ABTS of the BSs was high, similar to that of the BS-nanoARCs, and comparable to that of Trolox. The anti-inflammatory activity and the high antioxidant activity of the BSs, higher than that of YME itself, may be ascribed to the highly concentrated YME crown on the silver nanoparticle surface responsible for the observed SERS effect displayed by the BSs.

The lung microbiota can influence tumorigenesis; the genus *Pseudomonas* and *S. aureus*, among others, may negatively affect the outcome of an antitumor treatment [22,23,65]. The antimicrobial activities of BSs measured in µg Ag/mL were roughly 360–700-fold higher than those of YME, but were lost upon nebulization. The activities of the simplest hybrid formulation BS-nanoARCs instead endured nebulization, reducing the secretion of pro-inflammatory cytokines from THP-1 macrophages irritated with LPS, facilitating the resolution of bacterial lung inflammation, acting at the same time against *S. aureus*, an LPS-lacking Gram + microorganism, but displaying poor anti-biofilm formation and antifungal activities. The antimicrobial activity of silver nanoparticles depends on the release and further oxidation of Ag^0^ to Ag^+^ [66,67]. The structurally stable archaeolipid bilayers from the BS-nanoARCs probably impaired the release of Ag^0^, reducing its antimicrobial activity. In sum, freshly prepared nebulized BS-nanoARCs could exert simultaneous anti-inflammatory, antioxidant and partial antimicrobial activities. The cytotoxicity of nebulized [BS+ BS-nanoARCs] on A549 cells was also exerted if freshly prepared. Despite preliminary expectations, this study suggests that a combination of BS-nanoARCs and of [BS + BS-nanoARCs] (with BSs loaded in and on the outer surface of the nanoARCs and not simply mixed with the nanoARCs) may provide simultaneous cytotoxicity on A549 cells, anti-inflammation and antimicrobial activities. This study arose from noticing that most of the reports on nebulized nanoparticles were limited to assessing the structure of freshly prepared samples (without inspecting their activity after storage). However, revealing the mechanism behind BS and (BS + BS-nanoARC) cytotoxicity and limited storage and nebulization stability was outside the scope of this preliminary work. During storage, silver nanoparticles can dissolve, aggregate, modify their shapes or alter their molecular capping [68]; some of these changes can be detected by measuring their plasmonic peak position (shifted to longer wavelengths as their size increases, new peaks emerging in the near infra-network, among other changes [69]). In our case, after 30 days of storage in darkness at 4 °C, the size and plasmonic peak position of the BSs remained unchanged, but their cytotoxicity was lost. The data in this manuscript are insufficient to explain this phenomenon.

The mechanism of BS cytotoxicity after endocytosis, for example, includes different phenomena, such as the dissolution and oxidation from Ag^0^ to Ag+ in acid media (an effect linked to their deleterious effect on eukaryotic cells) [70] occurring in the presence of polyphenols refractory to acidity on the BSs’ surface [71]. The cytotoxicity of other silver nanoparticles (different sizes and capping) on A 549 cells has been ascribed to the loss of lysosomal activity [21]. After BS endocytosis, however, the fluorescence of Lysotracker RED was not reduced, meaning that BSs would not disturb the lysosomal activity of A549 cells. Distinct mechanisms, including ultrastructure destruction, ROS induction, DNA damage, enzyme inactivation and the regulation of signaling pathways have been described to mediate silver nanoparticle cytotoxicity on tumor cells [72,73,74,75]. It could be speculated that a single endocytic event of (BS + BS-nanoARCs) would internalize a greater number of BSs than the endocytosis of BSs alone; in such a case, the endocytosis of (BS + BS-nanoARCs) may magnify the above-mentioned mechanisms. Such an argument, however, does not explain the dependence of cytotoxicity on the distribution of BSs in (BS + BS-nanoARCs). Future studies employing powerful physical techniques such as those used in Villanueva et al., 2025 [76] will be necessary to describe in depth the structural features of hybrid nanoARCs.

The instability of (BS + BS-nanoARCs) to storage was also detected by a loss of cytotoxicity upon 30 days of storage in the dark at 4 °C. Such a loss could be related to a change in their structure (their size remained constant) due to BS dissolution, or dissociation from the nanoARC bilayers (since BSs partition into archaeolipid bilayers by weak non-covalent interactions). If so, the potential detachment of BSs could be hindered by incorporating cationic molecules (lipids or polymers) into the lipid bilayers to add ionic attractions between the bilayers and BSs. Pegylation using expensive pegylated lipids, on the other hand, increases the structural stability of classical liposomes [77], and not only would it not be necessary to increase the structural integrity of nanoARCs against aggregation, but it would likely reduce interaction with BSs [78].

## 4. Materials and Methods

### 4.1. Materials

Commercial yerba mate Rosamonte was used to prepare the YME. Soybean phosphatidylcholine (soyPC, Phospholipon 90 G, purity greater than 90%) was a gift from Phospholipid/Natterman, Ludwigshafen, Germany. 6-hydroxy-2,5,7,8-tetramethylchroman-2-carboxylic acid (Trolox), 2,2′-Azino-bis(3-ethylbenzothiazoline-6-sulfonic acid) (ABTS), methylthiazolyltetrazolium bromide (MTT), β-mercaptoethanol, phorbol 12-myristate 13-acetate (PMA), gallic acid and chlorogenic acid (CGA) were acquired from Sigma-Aldrich, Argentina. Pyruvate, L-glutamine, penicillin, streptomycin sulfate, trypsin, minimal essential medium (MEM) and Roswell Park Memorial Institute medium (RPMI) were acquired from Gibco, Buenos Aires, Argentina. Fetal bovine serum (FBS) was obtained from Internegocios, Córdoba, Argentina. Silver nitrate (AgNO_3_), Tween 80 and crystal violet were acquired from Biopack PA. All other reagents were of analytical grade and were acquired from Anedra, Argentina.

### 4.2. Synthesis and Characterization of Biogenic Silver Nanoparticles (BSs)

The synthesis of BSs was carried out using YME (Figures S6 and S7) as a reducing and stabilizing agent, according to Galdopórpora et al., 2021 [50]. Briefly, 10 mL of 10 mM AgNO_3_ was placed in test tubes, the pH was adjusted to 10 by adding 50 µL of NH_4_OH 50% *v*/*v* and it was heated at 100 °C, protected from light in a water bath. Subsequently, 160 µL of 150 mg/mL YME was added under stirring and incubated for 10 min until the reduction reaction was finished. Then, 200 µL of YME was added to induce the polyphenol coating on the BSs’ surface. Silver nanoparticle formation was detected spectrophotometrically by the presence of the plasmonic peak (λ_max_~427 nm [79]) by employing a spectrophotometer UV/Vis Shangai 9000S, Metash Instrument Co., Ltd., Shangai, China.

### 4.3. Oxidation State and Quantification of Ag in BSs by X-Ray Absorption

The Ag oxidation status in the BSs was determined by X-ray absorption near edge structure (XANES) and extended X-ray absorption fine structure (EXAFS) in an R-XAS looper Rigaku. Measurements were made in transmission geometry at room temperature, using an Ar ionization chamber as a detector for incident radiation and a semiconductor detector for transmitted radiation. The high-voltage and current conditions of the X-ray tube were set to 32 kV and 40 mA, respectively. A Si monochromator (400) was used to perform an energy scan between 25.4 and 26.1 keV. To compare the measurements, Ag^0^ and Ag_2_O were used as references under the same conditions.

### 4.4. Raman Spectra of BSs

The Raman spectra of BSs in aluminum-foil baskets were obtained employing i-Raman BWS415-532, BWTEK equipment with a 532 nm laser at 70% power, using an acquisition time of 10,000 ms (5 reps).

### 4.5. Preparation and Structural Characterization of Hybrid nanoARCs

Hybrid nanoARCs were prepared employing the thin-film hydration method. Briefly, 10 mg polar archaeolipids (PA), 30 µg BR extract and 4 mg Tween 80 (T80) dissolved in CHCl_3_:CH_3_OH (1:1 *v*/*v*) were poured into 2 mL microcentrifuge tubes. The organic solvents were evaporated in a gentle stream of N_2_ until a lipid film was formed. Subsequently, the lipid films were hydrated with 1 mL of BS suspension (~1 mg Ag/mL) (~10 mg lipids/1 mg Ag) under mechanical agitation with a magnetic diver and glass beads. The mean size and lamellarity were then reduced by bath sonication for 60 min at 80 W ultrasonic power and 40 Khz frequency. [BS + BS-nanoARCs] were prepared by adding aliquots from ~1 mg Ag/mL up to a final concentration of 12 or 25 µg Ag/mL for BS-nanoARCs.

BS-nanoARCs and unloaded BSs were separated via gel filtration on a Sephadex G-50 using spin columns. The eluates were seeded on a silica gel 60 thin-layer chromatography (TLC) plate (Merck F-254, 12 × 3 cm; thickness 0.2 mm) and developed using n-butanol, acetic acid and distilled water (8:2:2 *v/v*) as a mobile phase [80]. After chromatographic development, the plate was dried at 60 °C for 10 min and was then sprayed with 0.3% ninhydrin and dried at 45 °C for 5 min. Finally, the spots were observed under UV light.

*Size and ζ potential* were determined by dynamic light scattering (DLS) and phase analysis light scattering (PALS), respectively, using a nanoZsizer (Malvern Instruments, Malvern, UK). Samples were diluted 1:20 in Tris buffer (50 μL of samples with 950 μL of Tris-HCl buffer) for size and ζ potential determinations.

*Phospholipids and bacterioruberin quantification:* The colorimetric phosphate microassay was used to quantify phospholipids (PL) [81]. BR was quantified by absorbance at 490 nm using an average mass extinction coefficient of 2660 mL.mg^−1^. cm^−1^ [82].

*Transmission electronic microscopy (TEM):* Aqueous suspensions of BSs and BS-nanoARCs were deposited on a carbon grid, a drop of phosphotungstic acid (0.5% *w*/*v*) was poured on samples for 1 min and then the grids were air-dried and analyzed using a Transmission Electronic Microscope Jeol 1200-EX II (JEOL, Tokyo, Japan).

*Generalized polarization (GP) and fluorescence anisotropy (FA) of Laurdan*: The order and fluidity of the BS-nanoARCs were assessed by determining Laurdan GP and FA, respectively, using a LS55 Fluorescence Spectrometer [83]. To that aim, nanoARCs and BS-nanoARCs were labeled by incubation with Laurdan in Tris buffer at a 1:500 (for GP) or 1:20 (for FA) Laurdan/lipids (mol:mol) ratio for 30 min at room temperature.

GP was calculated using the following equation:

GP= (I440 − I490)/(I440 + I490)

where I440 and I490 are the fluorescence intensities at λem = 440 nm and λem = 490 nm, respectively, and obtained from the spectra between 400 and 520 nm at λex = 364 nm (Slitex: 5.0 nm and Slitem: 10.0 nm. Scan speed: 100 nm/min).

The fluorescence spectrometer software calculated FA according to the following equation:

FA= (I0 − G I90)/(I0 + 2G I90)

where I0 and I90 are the fluorescence intensities at λem = 440 nm with λex = 364 nm and the excitation polarizer oriented at 0° and 90°, respectively. The correction factor (G) was obtained from the ratio of emission intensity at 0° and 90° with the excitation polarizer oriented at 90° (after subtraction of scattered light).

### 4.6. Antioxidant Activity by ABTS Assay

ABTS radical cation (ABTS•+) was produced by mixing 500 µL ABTS stock solution (7 mM) with 800 µL of 4 mM potassium persulfate and allowing the mixture to stand in the dark at room temperature for 12–16 h before use. Then, the ABTS•+ solution was diluted with methanol up to an absorbance of 0.70, measured at 734 nm in a Metrolab 330 spectrophotometer. Samples were diluted at two-fold serial dilutions with methanol and 10 μL was mixed with 1 mL of diluted ABTS•+ solution in the dark at 30 °C for 30 min, then absorbance was measured at 734 nm. The radical scavenging activity (RSA) (%) was calculated as follows: (Abs negative control -Abs sample)/Abs negative control x 100, where the Abs negative control is the absorbance of 1ml of ABTS•+ and 10 μL of methanol, and the Abs sample is the absorbance of the tested sample. A calibration curve of Trolox (1.2–23 μg/mL) was prepared. The inhibitory concentration of the samples providing a 50% reduction in the radical scavenging activity (IC_50_) was calculated using Prism (GraphPad 8.0) from a dose–response-inhibition curve obtained by plotting the % RSA against concentration.

### 4.7. Antibacterial Activity

(i) Bacterial strains and growth conditions

Glycerol stocks of *Pseudomonas aeruginosa PAO1*_CV_ clone were a kind gift from Claudio Valverde at the Universidad Nacional de Quilmes, Argentina, where it is routinely used as a reference strain. The PAO1_CV_ lineage, derived from the PAO1DH clone used for decades in the laboratory of Prof. Dieter Haas (University of Lausanne, Switzerland) and *Staphylococcus aureus* (American Type Culture Collection (ATCC) 25923 strain) were stored at −80 °C. Frozen stocks were plated on lysogeny broth agar, grown for 24 °C and kept at 4 °C until use. For each assay, an overnight culture from a colony of plated stock of each bacterium was grown in lysogeny broth in a shaking incubator at 37 °C and 200 rpm for 18 h.

(ii) Anti-planktonic activity. Minimum inhibitory concentration (MIC)

MIC was determined by employing the broth microdilution assay following the Clinical and Laboratory Standards Institute recommendations (CLSI, 2018) [84]. Samples were diluted (two-fold serial dilutions) in cation-adjusted Mueller–Hinton broth (CAMHB) media and 80 µL were added to 96-well microplates. Subsequently, 20 µL of the bacteria culture diluted in CAMHB to obtain an inoculum containing approximately 1 × 10^7^ colony-forming units (CFU)/mL were added to each well. After 24 h of incubation at 37 °C, optical density (OD) was measured at 600 nm using a Cytation™ 5 Cell Imaging Multi-Mode Reader. The percentage of bacterial growth was calculated for the growth of the control in CAMHB as follows: % growth = (OD_sample_/OD_CAMHB_) × 100; where OD_sample_ is the OD after the incubation of bacteria with samples, and OD_CAMHB_ is the OD after the incubation of bacteria with the CAMHB. MIC_90_ was defined as the lowest concentration of samples in which 90% of growth is inhibited, using CAMHB as control [85].

(iii) Biofilm-formation inhibition

Biofilm-formation inhibition was determined according to Peeters et al., 2008 [86]. Briefly, samples were diluted (two-fold serial dilutions) in CAMHB media and 80 µL were added to 96 wells microplates. Subsequently, 20 µL of the bacteria culture diluted in CAMHB to obtain an inoculum containing approximately 1 × 10^7^ colony-forming units (CFU)/mL were added to each well. After 24 h of incubation at 37 °C, the supernatants were removed and the biofilm biomass was determined by crystal violet (CV) staining. Briefly, after two washes with phosphate buffer pH 7.4 (PBS) the plate was dried at 37 °C and the biofilm was fixed with methanol for 15 min. The methanol was then removed, the plate dried, and 0.1% *w*/*v* CV was added. After 20 min, two washes were carried out with distilled H_2_O, and the plate was dried again. The residua were subsequently solubilized with 30% *v*/*v* acetic acid and CV absorbance was measured at 595 nm (OD_CV_). The percentage of biofilm inhibition was calculated as follows: 100 − (OD_CVsample_/ OD_CVCAMHB_ × 100), where OD_CVsample_ was OD_CV_ after the incubation of bacteria with the sample and OD_CVCAMHB_ was OD_CV_ after the incubation of bacteria with CAMHB. The minimal biofilm disruption concentration (MBDC) was defined as the concentration required to reduce ≥90% of the biomass of the bacteria in the biofilm with respect to the non-treated biofilm.

### 4.8. Antifungal Activity

(i) Minimum inhibitory concentration (MIC)

To determine the yeast MIC, the microdilution method in broth recommended by CLSI (2017) was employed [84]. The inoculum of *Candida albicans* ATCC 10231 was prepared and adjusted to 5 × 10^3^ yeasts/mL by Neubauer chamber counting. A total of 100 μL of serial dilutions in Sabouraud-Glucose 2X medium (Sb-Glu 2X; Merck, Darmstadt, German) of samples was added to each well of a 96-well microplate, followed by 100 μL of inoculum suspension or 100 μL of sterile distilled water. As growth control (GC), culture medium with inoculum was used in the absence of formulations; as sterility control (SC), culture medium with sterile distilled water instead of inoculum was used in the absence of formulations; and as formulation control (FC), culture medium with formulations and sterile distilled water instead of inoculum was used. The plates were incubated in darkness for 24 h at 28–30 °C using a wet chamber. After incubation, the absorbance of the wells was read with a microplate reader at 405 nm. Each trial was conducted in triplicate. From these values, the% inhibition of fungal growth at the different concentrations tested was calculated. MIC was defined as the lowest concentration at which 100% growth inhibition was observed.

(ii) Minimum fungicidal concentration (MFC)

To determine the MFC, 10 μL from the well containing a concentration higher than or equal to the MIC was taken from microplate and seeded in a Petri dish containing Sb-Glu 2X. The fungal growth was observed after incubation in darkness at 28–30 °C. MFC was defined as the minimal concentration in which no growth was observed.

### 4.9. Cell Lines, Culture Conditions and Cytotoxicity

Human epithelial lung cell line A549 (ATCC^®^ CCL-185™) was maintained in MEM supplemented with 10% FBS, 100 U/mL penicillin, 100 μg/mL streptomycin and 2 mM L-glutamine. The human monocyte cell line THP-1 (ATCC TIB-202™) was a gift from Dr. Jessica Minnaard (Centro de Investigación y Desarrollo en Criotecnología de Alimentos, CIDCA. Universidad Nacional de La Plata) and was maintained in RPMI supplemented with 100 U/mL penicillin, 100 μg/mL streptomycin, 2 mM L-glutamine, 0.05 mM 2-mercaptoethanol and 1 mM sodium pyruvate. THP-1 monocytes were differentiated into macrophages by treatment with 100 ng/mL PMA for 24 h. All cell lines were grown in a humidified atmosphere of 5% CO_2_ at 37 °C.

The viability of THP-1 macrophages or A549 treated with BSs or hybrid nanoARCs was measured by an MTT assay [87]. Briefly, THP-1 macrophages were seeded in 96-well plates at 2 × 10^4^ cells per well and A549 cells were seeded at 1 × 10^4^ cells per well and both were grown for 24 h. Then, the cells were incubated with 100 μL fresh medium supplemented with 5% FBS containing BSs (6, 12.5, 25, 50, 100 and 200 µg /mL Ag); BS-nanoARCs (6–0.6; 12.5–1.2; 25–2.5; 50–5; 100–10; 200–20 µg/mL PL-µg/mL Ag, respectively; and [BS + BS-nanoARCs] (50 µg/mL PL + 25/ 50 µg/mL BS). After 24 h (for THP-1 cells) or 72 h (for A49 cells) of incubation, the cells were washed with PBS and 110 μL of 5 mg/mL MTT solution was added to each well. After 3 h of incubation, the MTT solution was removed, the insoluble formazan crystals were dissolved with 150 μL of ethanol and absorbance was measured at 570 nm in a microplate reader (Cytation™ 5).

### 4.10. Anti-Inflammatory Activity on THP-1 Macrophages

The in vitro anti-inflammatory activity of the BSs and hybrid nanoARCs was determined by measuring the release of IL-6, IL-8 and TNF-α by cells stimulated with LPS. Briefly, THP-1 macrophages were seeded at 2 × 10^5^ cells per well into 24-well plates and grown for 24 h. Then cells were co-incubated with 1 µg/mL LPS and BSs or BS-nanoARCs (50 µg PL/mL; 5 µg Ag/mL), or control samples. LPS-stimulated and non-stimulated cells without treatments were used as positive and negative controls, respectively. After 24 h of incubation, supernatants were collected and stored at −20 °C until analysis. Human TNF-α, IL-6 and IL-8 levels were measured by an enzyme-linked immunosorbent assay (BD OptEIA^TM^, BD Biosciences, San Jose, CA, USA) following the manufacturer’s instructions. Absorbance measurements were carried out at 450 nm on a microplate reader.

### 4.11. Intracellular Reactive Oxygen Species (ROS) Production

The capacity of BSs or BS-nanoARCs to reduce the generation of intracellular ROS on human macrophages stimulated with LPS was measured with carboxy-H_2_DCFDA dye. Briefly, THP-1 macrophages were seeded at 3 × 10^4^ cells per well into 96-well plates and grown for 24 h. Then cells were co-incubated with 1 µg/mL LPS and BSs, BS-nanoARCs (50 µg PL /mL; 5 µg Ag /mL) or control samples in fresh medium with 5% FBS. After 24 h incubation, cells attached at the bottom of the well were washed twice with PBS and incubated with a solution of carboxy-H_2_DCFDA for 30 min at 37 °C. LPS-stimulated cells were used as a reference. The fluorescence intensity of whole cells was measured using Cytation^TM^ 5 equipment (ʎ_excitation_ 490 nm and ʎ_emission_ 520 nm.

### 4.12. Effect on Lysosomes upon Uptake by THP-1 Macrophages and A549 Cells

The effect on lysosomes after uptake of BSs or BS-nanoARCs by THP-1 macrophages and A549 cells was tracked with the fluorescent acidotropic probe LysoTracker ™ Red DND-99 (LR). Briefly, THP-1 macrophages were seeded on 24-well plates at a density of 1.5 × 10^5^ cells per well with rounded coverslips at the bottom and allowed to attach for 24 h. Then, cells were incubated with BSs, BS-nanoARCs (50 µg PL /mL; 5 µg Ag /mL) or control samples in MEM supplemented with 5% FBS for 6 h at 37 °C. After incubation, supernatants were discarded and cells were washed with PBS and incubated with 3.8 µM LR and 3.2 µM Hoechst for 30 min at 37 °C. Afterward, the cells were washed 5 times with PBS and fixed for 5 min with 3.75% formaldehyde *w*/*v* in PBS. Finally, the preparations were washed three times in PBS and mounted in a 90% *w*/*v* glycerol solution onto a slide. Confocal microscopies were obtained with a Leica laser-scanning spectral confocal microscope TCS SP8 (Leica Microsystem, Wetzlar, Germany) using 577/590 nm and 350/461 nm excitation/emission lasers for LR and Hoechst, respectively.

### 4.13. Stability to Light, Temperature and Nebulization Stress

The colloidal stability of BSs and hybrid nanoARCs was determined by measuring size, ζ potential, and plasmonic peak wavelength after storage at 4 °C in the dark for 30 days or being submitted to direct light incidence (samples placed on transparent tubes at 50 cm of an 18W tube lamp light (λ > 420 nm) at 20 °C). After that, the cytotoxicity on A549 cells was determined as stated in Section 4.9.

The stability of BSs and hybrid nanoARCs freshly prepared and after storage for 30 days at 4 °C in the dark to nebulization stress was screened using a vibrating mesh nebulizer (Omron NE-U22, OMRON Healthcare, Kyoto, Japan) according to Altube et al., 2020 [88]. Briefly, 2 mL of BSs (200 µg Ag /mL) or hybrid nanoARCs (200 µg PL/mL) were nebulized for 5 min and the aerosols were collected in a vessel connected to the nebulizer; the lipid recovery was determined by PL quantification, size, ζ potential, plasmonic peak wavelength and cytotoxicity on A549 was determined (BSs at 12, 25 and 50 µg /mL Ag; and [BS+ BS-nanoARC] at 50-12 µg /mL PL µg /ml Ag respectively)

### 4.14. Statistical Analysis

Statistical analyses were performed using the Kruskal–Wallis non-parametric test followed by Dunn’s multiple comparison procedure using Prisma 8.0.2 Software (Graph Pad, San Diego, CA, USA). Differences were considered statistically significant at a *p*-value of <0.05. * *p* < 0.05; ** *p* < 0.01; *** *p* < 0.001; **** *p* < 0.0001; n.s. represents not significant (*p* > 0.05).

## 5. Conclusions

Alongside antitumoral agents, nebulized hybrid nanoARCs may constitute alternatives to corticosteroids and antibiotics, aiding in reducing the problem of environmental antibiotic waste in human or veterinary fields. Despite their simple manufacturing, their lability upon storage should be corrected in future approaches, since it constitutes a main limiting factor for their use as therapeutic agents.

## Figures and Tables

**Table 1:** Structural features of formulations.

Formulation	Z-Average (nm ± SD)	PDI ± SD	ζ Potential (mV ± SD)	GP	FA
BS	78 ± 43	0.44 ± 0.07	−28 ± 1.0		
nanoARC	444 ± 206	0.58 ± 0.13	−55 ± 3.2	−0.06	0.21
BS-nanoARC	95 ± 37	0.61 ± 0.16	−58 ± 5.5	−0.26	0.21
[BS + BS-nanoARC]	176 ± 50	0.47	−38 ± 0.6		

Values are expressed as mean ± standard deviation (SD) (*n* = 7). PDI = Polydispersity index.

**Table 2:** Antioxidant activity of formulations measured by ABTS.

Formulation	IC50 (μg/mL)		
	Ag	YME	PL
BS	5 ± 1	-	-
BS-nanoARC	4 ± 2	-	24 ± 17
YME	-	21 ± 19	-
nanoARC	-	-	>58 ± 37

**Table 3:** IC_50_ values of formulations on THP-1 macrophages.

Formulation	IC50 (μg/mL)		
	Ag	YME	PL
BS	21.7	-	-
YME	-	>160	-
BS-nanoARC	6.9	-	70
nanoARC	-	-	133.2

**Table 4:** Minimum inhibitory concentration (MIC) of formulations against planktonic *S. aureus* and *PAO1*, and minimal biofilm disruption concentration (MBDC) against biofilm formation.

Formulation	MIC (μg/mL)						MBDC (μg/mL)					
	*S. aureus*			*PAO1*			*S. aureus*			*PAO1*		
	Ag	YME	PL	Ag	YME	PL	Ag	YME	PL	Ag	YME	PL
BS	12.5	-	-	6.4	-	-	6	-	-	7.5	-	-
BS-nanoARC	13	-	130	50	-	500	83	-	830	83	-	830
YME	-	4500	-	-	4500	-	-	2300	-	-	2300	-
nanoARC	-	-	>1000	-	-	>1000	-	-	>1000	-	-	>250

**Table 5:** Minimum inhibitory concentration (MIC) and minimum fungicidal concentration (MFC) of formulations against *C. albicans* ATCC 10231.

Formulation	MIC (μg/mL)			MFC (μg/mL)		
	Ag	YME	PL	Ag	YME	PL
BS	12.5	-	-	25	-	-
BS-nanoARC	50	-	500	>100	-	>1000
YME	-	>1000	-	-	>1000	-
nanoARC	-	-	>1000	-	-	>1000

**Table 6:** IC_50_ values of formulations on A549 cells.

Formulation	IC50 (μg/mL)		
	Ag	YME	PL
BS	35.9	-	-
YME	-	>160	-
BS-nanoARC	20	-	200
[BS + BS-nanoARC]	12	-	50
nanoARC	-	-	>200

**Table 7:** Structural features of BSs and hybrid nanoARCs freshly prepared (day 0) and 30 days after storage in the dark at 4 °C pre- (−) and post-nebulization (+).

Formulation	Days of Storage	Nebulization	Z-Average (nm)	PDI	ζ Potential (mV)	ABTS IC50 (μg Ag/mL)	% PL Recuperation After Nebulization
BS	0	−	59	0.452	−34.9	7	−
	30	−	74	0.493	−35.1	7	−
	0	+	56	0.428	−25.6	−	−
	30	+	59.95	0.929	−25.3	−	−
BS-nanoARC	0	−	68	0.436	−44	6.1	−
	30	−	144.6	0.722	−65.4	6.4	−
	0	+	79	0.389	−42.1	−	40
	30	+	>1000	1	−38	−	40
[BS + BS-nanoARC]	0	−	190	0.451	−39	7	−
	30	−	125.4	0.506	−66.2	6.5	−
	0	+	200	0.422	−32.5	−	30
	30	+	>1000	1	−66.2	−	30

## Data Availability

Data are contained within the article.

## Supplementary Materials

The following supporting information can be downloaded at: https://www.mdpi.com/article/10.3390/ijms26010392/s1. References [89,90,91,92] are cited in the Supplementary Materials.
