# Supplementary material for: Nebulized Hybrid Nanoarchaeosomes: Anti-Inflammatory Activity, Anti-Microbial Activity and Cytotoxicity on A549 Cells
Source: Int J Mol Sci. 2025 Jan 4;26(1):392. doi: 10.3390/ijms26010392 (PMC11721710; doi:10.3390/ijms26010392)
Supplement: Supplementary file 1 [file ijms-26-00392-s001.zip › ijms-3396619-supplementary.pdf]

Supplementary material

Materials and methods and Results

S1. Archaea growth, archaeolipids extraction and characterization

Halorubrum tebenquichense archaeas were grown in 15 l basal medium supplemented with yeast extract and glucose at 40 ◦C and 600 rpm in a home-made bioreactor and harvested after 96 h growth. PA were extracted using the Bligh and Dyer method modified for extreme halophiles; dried and stored at - 20 ◦C until used. PA were routinely screened by phosphate content [81] and electrospray-ionization mass spectrometry. The BR extraction was carried out according to Higa 2020 [89]. BR was characterized by UV–visible spectra (300–700 nm) and quantified by absorbance at 490 nm using an average extinction coefficient of 2660 $ml.mg^{-1}$. cm^{-1} [82].

S2. Preparation and characterization of yerba mate *[Ilex paraguarensis]* extract (YME)

Ilex paraguariensis (yerba mate), a plant belonging to the *Aquifoliaceae* family whose dried leaf infusion (a tea-like beverage, the *mate)* is typically consumed in Argentina, Paraguay, Uruguay and Brazil [90] with antimicrobial, antioxidant anti-inflammatory and antitumoral properties ascribed to its polyphenols content, particularly to its high content on 3-O-caffeoyl quinic acid or chlorogenic acid (CGA) and its isomers [91]. Commercial Rosamonte *Ilex paraguariensis* leaves and stems were ground using an Ultra-Turrax IKA homogenizer and mixed at 1 g / 7 ml with 1:1 v/v acetone: water solution. After stirring at 120 rpm at 50°C for 30 min, the suspension was filtered four times with gauze and finally Whatmann 28 µm filter paper. The eluate was vacuum dried in a desiccator to constant weight and stored at 4°C until use. The mass of yerba mate solid extract was expressed as a function of the initial yerba mate mass [92]. The YME was prepared by dissolving 180 mg of the solid in 1 ml buffer Tris or MilliQ water and heated at 100°C for 60 min. After extraction, a YME was obtained (Figure S6A).

S3. Total phenol content in YME

The total phenol content (TPC) was assessed using the Folin-Ciocalteu (FC) method. The content was expressed as gallic acid equivalents using a gallic acid (GA) (0-30 µg GA/ml) standard curve. YME was diluted with water at 1:2 ratio. Briefly, 12.5 µL of extract (5 mg yerba mate solid/ml) with 62.5 µL (1:10 v/v) FC reagent in a 96-well-plate for 5 min at room temperature in the dark, and further adding 50 µl of 7.5% w/v Na_2CO_3 solution and mixing. After that, the absorbance was measured at 760 nm using a Cytation 5 Cell Imaging Multi-Mode Reader. The results were expressed in gallic acid equivalents (GAE mg/1 g solid mass). The TPC assay was performed by triplicate (Figure S6 B and C).

S4. Chlorogenic acid content of YME (HPLC)

The CGA in YME was quantified by HPLC employing a Merck Chromolith RP18 (100 × 4,6 mm) (Darmstadt, Alemania) column, with a 321 Pump, 151 detector UV/Vis and 234 series autoinjector (Middleton, WI, USA). The run was carried out employing a binary gradient made of A: distilled water (0.072% trifluoracetic acid) / B: acetonitrile, as follows: 10 min A:B 97:3 v/v, 3 min A:B 65:35 v/v, 5 min A:B 65:35 v/v and back to initial conditions, at 0.85 ml/min flow rate at λ = 327 nm. A calibration curve of peak areas versus chlorogenic acid concentration (0-6 µg/ml) was plotted.

The dry residue of YME yielded 109 ± 5 µg gallic acid equivalents /mg of YME, corresponding to 73.4 µg CGA/ mg of YME (Figure S7 A, B).

S5. Determination of Ag Oxidation State in BS by RX Absorption

To determine the oxidation state of Ag in BS, X-ray absorption (XANES and EXAFS) was applied at the K-bordertags. Previously, BS were filtered sequentially by 1.20 µm nylon membrane, then 0.45 µm, and finally by 0.22 µm. Figure S2 shows the XANES spectra of the samples along with two references of Ag(0) and Ag_2O. The oscillations found after the absorption edge in the studied sample coincide with those typical of metallic Ag, although slightly more attenuated, the latter could be due to the nanostructured nature of the particles.

Figure S3 shows the magnitude of the Fourier transform of the EXAFS oscillation of the BS along with the references, which represents a pseudo-distribution of distances to the absorbing

atom. Here it can be seen how the BS presents the two characteristic peaks of the metallic Ag and that correspond to the Ag-Ag interaction in this structure. It is observed that these peaks are attenuated and slightly wider than those presented in the Ag reference, this could indicate that there is a degree of disorder in the Ag-Ag distances in the synthesized sample. In addition, the presence of a shoulder at approximately 1.4 Å (grey area Figure S3) is observed, which could indicate that a tiny fraction of the Ag atoms in the sample are oxidized (about 10%), due to the similarity between the distance found in this contribution and that present in the Ag_2O reference. This fraction of Ag_2O could be segregated from the rest of the BS, or present in the form of surface oxide on them. It is confirmed that most of the BS are as Ag^0, and there is a contribution that shows that there is some Ag_2O, which could be as a segregated phase that was oxidized, or as surface oxide in the BS.

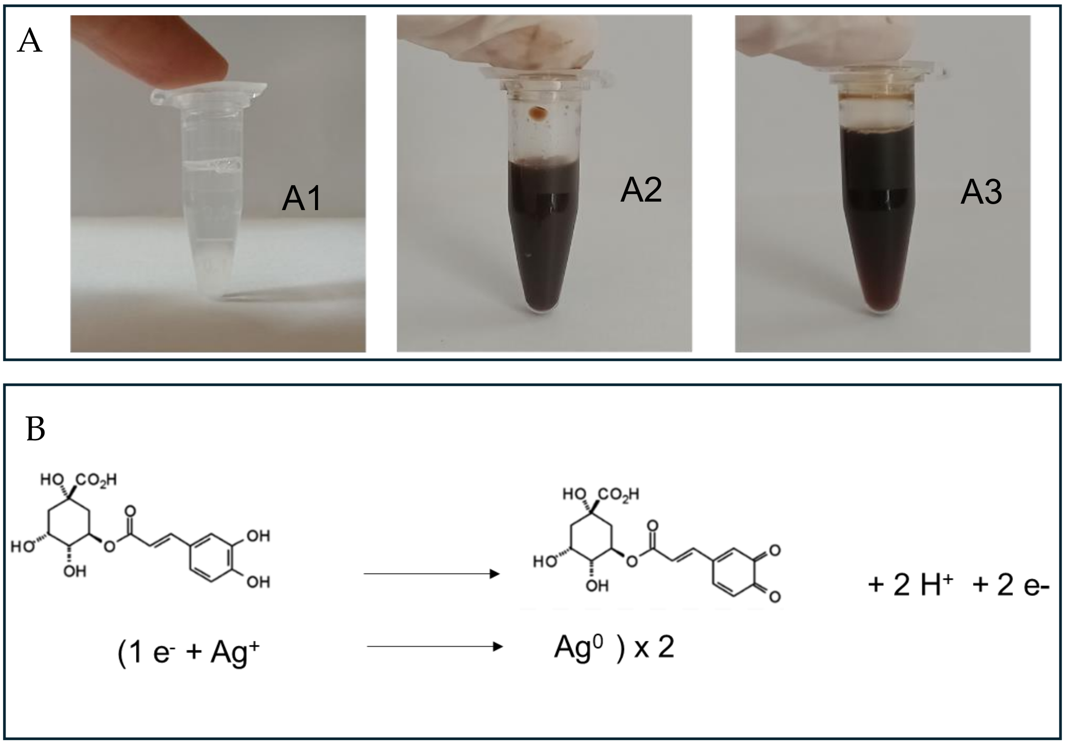

Figure S1. (A1) $AgNO_3$ solution; (A2) first addition of YME; (A3) second addition of YME; (B) representative redox between CGA and Ag+ leading to Ag^0.

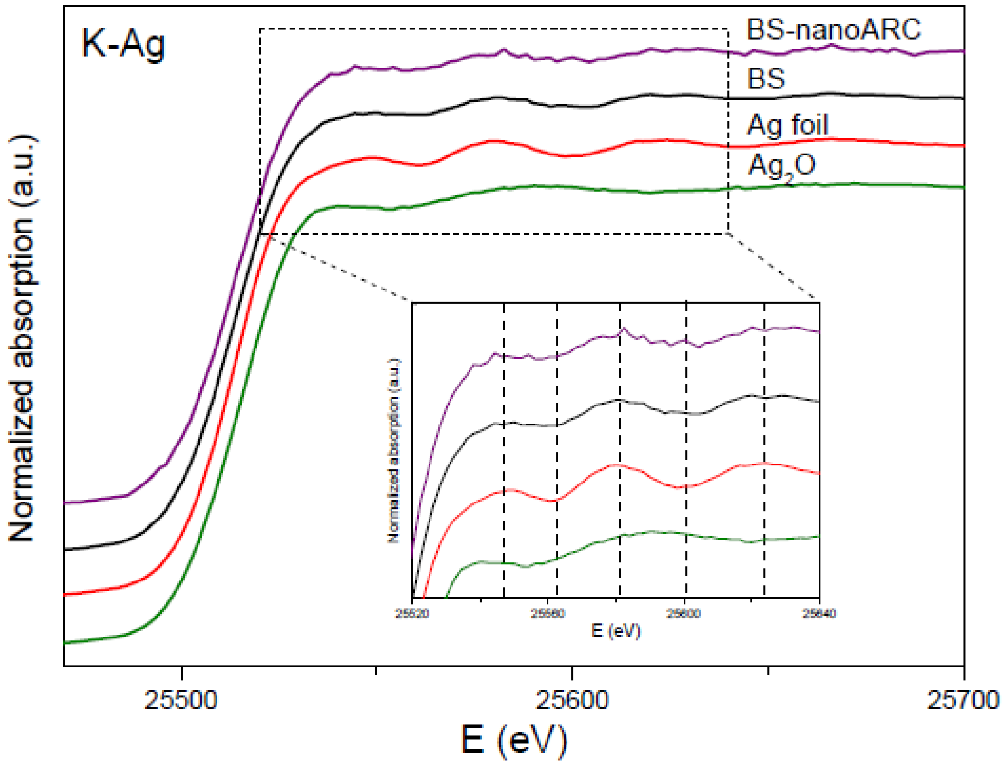

Figure S2. XANES spectrum of BSs, BS-nanoARCs, Ag foil and Ag_2O used as references of Ag^0 and Ag+, respectively.

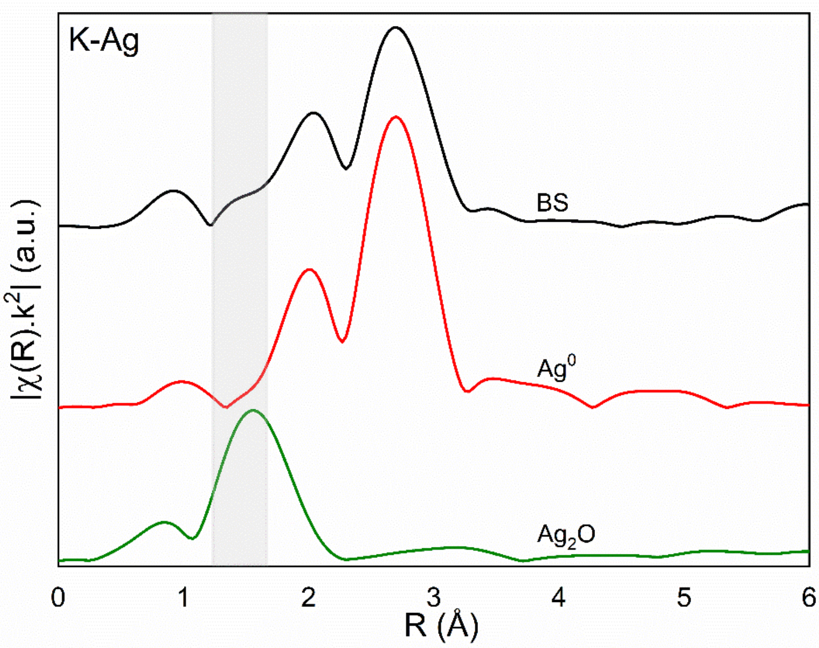

Figure S3. Fourier transform amplitude from EXAFS oscillation of BSs and of Ag foil and Ag_2O used as references of Ag^0 and Ag^+, respectively.

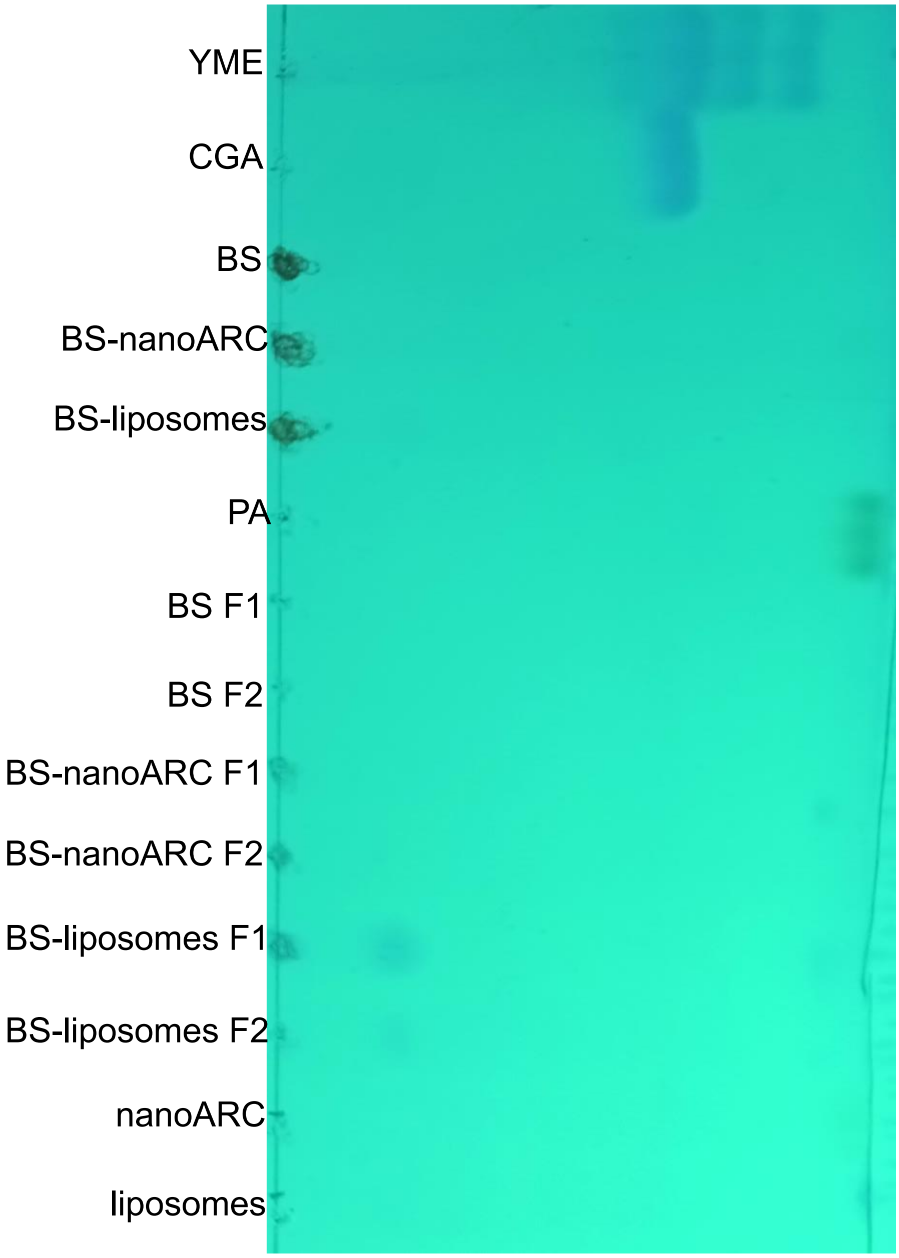

Figure S4. Thin-layer chromatography of formulations.

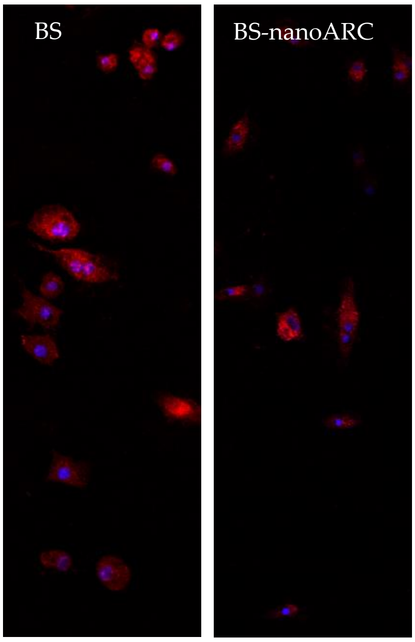

Figure S5. Confocal fluorescence images of A549 cells stained with Lysotracker (red) and Hoechst (blue) after incubation with BSs and BS-nanoARCs. Magnification 20×.

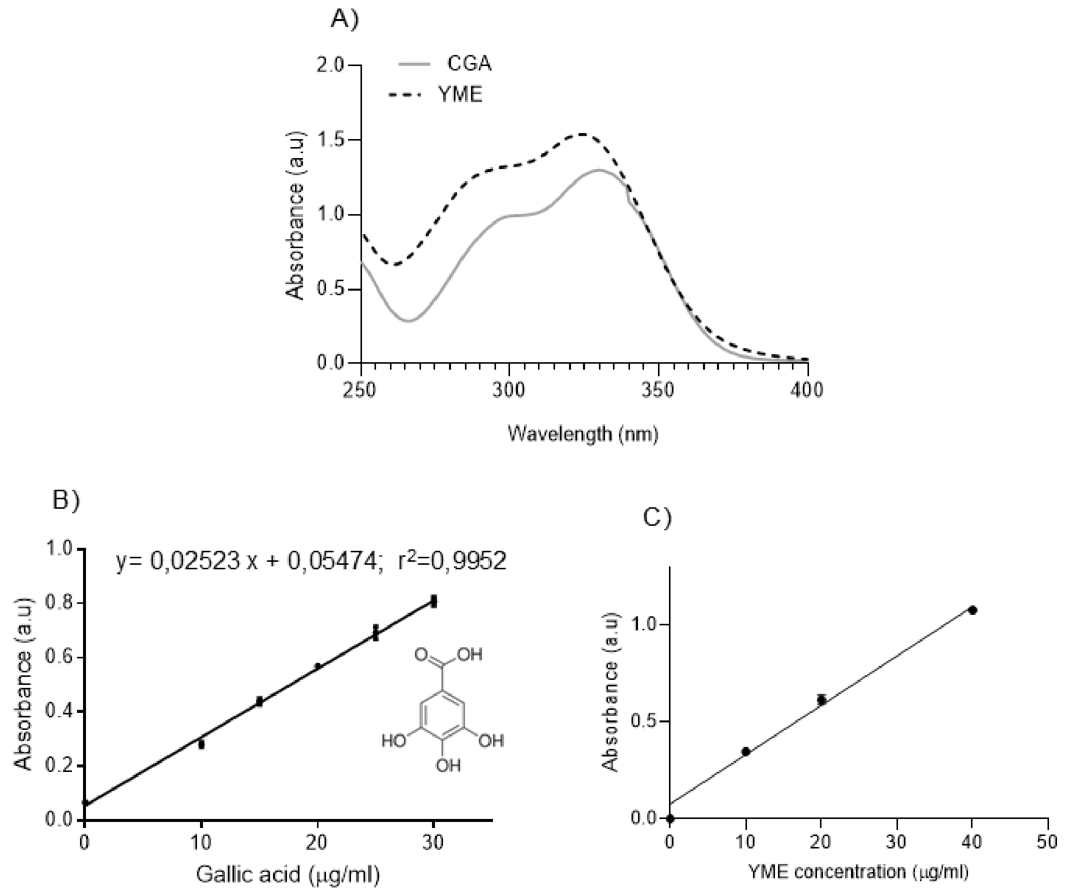

Figure S6. (A) Yerba mate extract (YME) and CGA spectra. Calibration curve for (B) gallic acid (lineal between 0 and 30 μg/mL) and (C) YME as measured by Folin Ciocolteau.

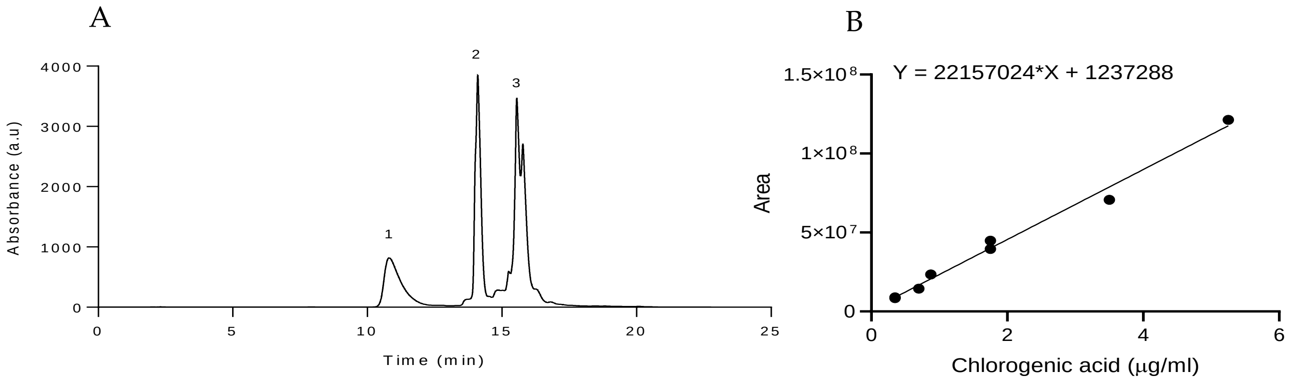

Figure S7. (A) A YME HPLC chromatogram: the CGA (3-o caffeoyl quinic acid) standard showed a peak at 14.15 min (peak 2). Given the reported low proportion of theobromine [91] and the absent absorption of caffeine at 325 nm, a previous report [91] suggests that the peaks at lower and higher retention times may correspond to the following CGA isomers: neochlorogenic acid (5-o caffeoyl quinic acid) (peak 1) and crypto chlorogenic acid (4-o caffeoyl quinic acid)/ caffeic acid (peak 3), respectively. (B) Calibration curve of standard CGA (lineal between 0 and 6 µg/mL).
